# The place of human rights and the common good in global health policy

**DOI:** 10.1007/s11017-016-9372-x

**Published:** 2016-08-01

**Authors:** John Tasioulas, Effy Vayena

**Affiliations:** 1 Dickson Poon School of Law, King’s College London, Somerset House East Wing, London, WC2R 2LS UK; 2 Epidemiology, Biostatistics and Prevention Institute, University of Zurich, Hirschengraben 84, 8001 Zurich, Switzerland

**Keywords:** Global health, Justice, Human rights, Right to health, Common goods, Public health

## Abstract

This article offers an integrated account of two strands of global health justice: health-related human rights and health-related common goods. After sketching a general understanding of the nature of human rights, it proceeds to explain both how individual human rights are to be individuated and the content of their associated obligations specified. With respect to both issues, the human right to health is taken as the primary illustration. It is argued that (1) the individuation of the right to health is fixed by reference to the subject matter of its corresponding obligations, and not by the interests it serves, and (2) the specification of the content of that right must be properly responsive to thresholds of possibility and burden. The article concludes by insisting that human rights cannot constitute the whole of global health justice and that, in addition, other considerations—including the promotion of health-related global public goods—should also shape such policy. Moreover, the relationship between human rights and common goods should not be conceived as mutually exclusive. On the contrary, there sometimes exists an individual right to some aspect of a common good, including a right to benefit from health-related common goods such as programmes for securing herd immunity from diphtheria.

What are the demands of justice applicable to global health policy? By global health policy we mean those practical measures, whether adopted and implemented by international organizations, states, corporations, or agents of some other kind, that have as their ultimate goal, in the words of the World Health Organization’s evocative motto, “health for all.” They are legal and other measures aimed at protecting and promoting the interest in health of every human being around the globe. In keeping with a long philosophical tradition, we deploy two distinct senses of the idea of justice. According to the first, justice concerns moral duties that are owed to and claimable by others as a matter of *individualrights*. In another, broader sense, justice concerns moral *duties* governing our conduct towards others, especially insofar as they fall within the proper remit of public decision-making.^1^ The second sense of justice, the domain of other-regarding moral duties, includes the first sense, the domain of justice as rights, as a component. But it also includes other moral duties, notably duties to preserve and promote the *common good* that may not be linked to rights.

Transposed to the global context, justice, to a significant degree, consists in the morality of individual human rights and global common goods. To this extent, a justice perspective on global health policy must be bifocal in character. However, we contend that it is a profound error, if also a common one, to construe the two strands of justice as being in an inherently dichotomous and generally antagonistic relationship. Not only do we need to draw on both human rights and common goods, but the ‘individualism’ of human rights is not to be starkly juxtaposed against the ‘collectivism’ of the common good. On the contrary, human rights are an integral component of some global common goods. This article seeks to make a start on elaborating the meaning and implications of such an integrated, bifocal perspective in relation to global health policy.^2^

We begin, in the first section, by outlining the distinctive character of human rights: they are moral rights possessed by all human beings simply in virtue of their humanity. Then, in light of the prominent role of human rights in global health policy debates, the next two sections focus on the human right to health. One important question is how that right is to be individuated within the overall set of human rights. Contrary to a popular, radically ‘inclusive’ interpretation, we suggest characterizing the human right to health’s scope of concern primarily by reference to obligations regarding health care services and public health measures. This way of understanding the human right to health makes it clear that it is only one among a number of human rights that serve our interest in health and to which global health policy needs to be responsive. We then offer an account of how to specify the content of the human right to health, i.e., the content of the duties regarding health care services and public health measures associated with the right. The process of content specification, we argue, involves the application of a threshold criterion that incorporates considerations of possibility and burden. In the fourth section, we explain why human rights cannot do all the work in shaping a just global health policy, giving special attention to the crucial role of health-related global common goods. We also respond to the converse hypothesis that global policy must be predominantly concerned with common goods *as opposed to* human rights. This response turns on showing how common goods may include, as a component, arrangements that secure human rights.

## Introducing human rights

Global health policy advocates have repeatedly called for a post-2015 development agenda that gives a prominent place to policy objectives couched in the language of human rights. These calls echo the chorus of agreement among a wide variety of international actors—including the United Nations, NGOs, governments, and ordinary citizens—on the vital importance of a human rights basis for the new development goals more generally [3–5]. Charitably interpreted, as more than just a rhetorical ploy intended to convey a sense of urgent commitment, this emphasis on human rights embodies a vital insight. The adoption of goals simply concerned with the promotion of human welfare—such as our interests in health, prosperity, education, etc.—is not enough. Human rights inject a distinctive moral dimension into policy objectives, one that is especially responsive to the plight of victims of injustice throughout the globe.

The distinctive character of human rights consists in the fact that they are universal moral rights: moral rights possessed by all human beings simply in virtue of their humanity.^3^ They mark the threshold at which each individual human being’s interests generate *duties* or *obligations* (we use these terms interchangeably) on the part of others to respect, protect, and promote those interests in various ways. The violation of an obligation is a moral wrong, whereas no wrong is committed simply by thwarting another’s interests or leaving those interests unpromoted. For example, neither beating a rival for a coveted job nor failing to donate your spare healthy kidney for a transplant need be wrongful. Human rights are a distinctive moral register of critical assessment, beyond assessments tracking rises and falls in individual or collective welfare. The foregoing does not mean that wellbeing as such, or elements of it such as the global burden of disease, lacks normative significance. It is just to say that it cannot displace the distinctive kind of moral assessment introduced by human rights: the idea of moral duties owed to each and every human being, the violation of which specifically victimizes the right-holder. Indeed, the discourse of human rights is at the core of a ‘global justice’ approach to health: justice, on one historically influential interpretation, consists in the rights-involving part of morality, and the sub-category of human rights are those moral rights that are held globally because they are possessed by people simply in virtue of their humanity.

So far, we have spoken of human rights as a certain kind of moral norm. Of course, there is now a firmly established doctrine of international human rights law in which various health-related human rights form an integral part.^4^ Moreover, in excess of two-thirds of national constitutions explicitly include health rights, often by incorporating provisions in international human rights treaties [2, p. 263]. But the morality of human rights is independent of its recognition by domestic or international law. A right does not need to be actually legally enshrined, let alone enforceable, to exist as a human right. On the contrary, human rights law is best understood as deriving its distinct identity from the attempt to give legal effect to background human rights morality, insofar as it is appropriate to do so. It is the background morality of human rights that is the main focus of this chapter. Three further preliminary observations are worth making in this connection.

First, the duties associated with human rights include positive duties to engage in certain forms of conduct, such as the provision of health care services, as well as negative duties to refrain from certain conduct, such as administering medical treatment without consent. Moreover, the positive duties associated with a right may be primary duties. In other words, they are duties that are not parasitic on other duties associated with the right, such as positive duties to compensate or make reparation triggered by a violation of some prior duty. Instead, human rights also impose primary positive duties to make certain goods and services available to their holders. Of course, there are special problems in the allocation of positive primary duties to duty-bearers, and in the specification of their content, which do not arise in the case of negative primary duties [8]. But these differences between the two kinds of rights, which are largely matters of degree, do not warrant the wholesale expulsion of so-called ‘socio-economic rights’, with positive primary duties, from the category of bona fide human rights [9].

Second, there is no compelling a priori reason why the duties associated with human rights should be thought to fall exclusively on states, at least as primary duty-bearers. This idea is a distortion that a misplaced focus on legal instruments—constitutions and treaties—has introduced into thinking about human rights. Instead, we should maintain an open-minded and flexible attitude to the question of who the relevant duty-bearers are in any given time and place [6, 10]. Multinational corporations, international organizations, and even individuals can be directly subject to human rights-related duties. Pharmaceutical companies, for example, may be directly subject to human rights obligations to make antiretrovirals and other drugs available to developing countries at a significantly lower cost than market price [11]. In an environment of accelerating globalization, with a concomitant decline of state power relative to various other global actors, the importance of not conceptually restricting human rights obligations to states is all the more pronounced. Indeed, precisely this insight is at the heart of the innovative UN Guiding Principles on Business and Human Rights, which seek to provide an authoritative specification of the human rights responsibilities directly applicable to corporations [12].

Finally, another a priori commitment to be resisted is the idea that a *pro tanto* case always exists for enshrining human rights as legal entitlements, let alone for taking the further step of making them enforceable legal entitlements [6, 13]. Law is a vitally important mechanism of implementation, but it remains one mechanism alongside others, including social conventions, public opinion, and the inculcation of a rights-respecting ethos through fostering the internalization of human rights norms by individual and collective agents. As Sen has stressed, whether and to what extent individual human rights should be enshrined in counterpart legal rights are a matter of what is inherently appropriate and works in all the circumstances, which is subject to considerable variation in time and place. Experience shows that making human rights legally claimable is sometimes counter-productive. For example, in Brazil the constitutionalization of the right to health appears to have facilitated a transfer of health resources to wealthier members of society who can afford the cost of litigation [14]. The overall health budget remained fixed, but the better-off engaged in litigation against the government to siphon off a larger share of it for themselves, often in order to treat less serious ailments.^5^ To take another example, the economist Jeffrey Sachs, one of the chief architects of the Millennium Development Goals (MDSs), has ascribed the success in meeting those goals partly to the fact that states are not legally bound by them. This lowered the cost of states publicly signing up to the goals in the first place, enhancing the likelihood that they would do so [16]. In short, the difficult and multi-faceted question of legalization is one that deserves extensive consideration on a case-by-case basis. No presumptive answer to it is already inscribed in the very nature of human rights.

## Individuation and inclusivity

Human rights exist insofar as universal human interests generate obligations on others to respect, protect, and promote those interests in various ways. Interests are here understood as the elements of wellbeing, the realization of which in a person’s life make it a better life for them. We favour an objectivist and pluralistic account of the interests that ground human rights [17]. They are interests human beings possess independently of whether they actually desire their realization, and they are not limited to one kind of interest—autonomy, for example—or to one category of interests, such as those interests that qualify as basic needs [18]. Instead, a plurality of genuinely universal human interests are capable of generating duties on the part of others in the case of all human beings, simply in virtue of their humanity. Moreover, essential to the rights-generative role of human interests is that they belong to distinct individuals with equal moral status in virtue of their humanity: the status of human dignity. This is central to explaining the resistance of human rights to trade-offs both against other rights and against non-rights based considerations.^6^

The pluralistic theory of human rights claims not only that a plurality of interests is relevant to the justification of human rights generally but also that any given individual human right is typically grounded in a plurality of interests, such as autonomy, health, knowledge, friendship, accomplishment, play, etc. [17]. The right not to be tortured, for example, is grounded not only in one’s interest in autonomy but also in one’s interest in being free from pain and in being able to form intimate and trusting relationships. This is also true of the human right to health: it serves not only one’s interest in health but also various other interests that enjoying good health can enable one to realize, such as making friends, acquiring understanding, or accomplishing something with one’s life. Indeed, the right to health may even include entitlements to medical services, such as non-therapeutic abortions or cosmetic surgery, that are not primarily intended to serve the health interests of the right-holder. Hence, a diversity of interests helps to justify the existence of a human right to health and to shape its associated obligations.

One way to fall into the trap of assuming that the human right to health is grounded exclusively in our interest in health is to adopt an unduly expansive interpretation of health. This is precisely what the WHO did in the preamble to its constitution, which notoriously states that ‘health is a state of complete physical, mental and social well-being and not merely the absence of disease and infirmity’ [20]. But, as has been repeatedly shown, this definition is far too broad. Health, on any remotely useful understanding, is one element of wellbeing among others, not the whole of it. And this remains the case even though health bears pervasive constitutive and instrumental relations to the other elements of wellbeing. For the purposes of this article, we take health to be centrally concerned with the effective functioning of standard human physical and mental capacities [21]. A person can enjoy such functioning even when they are deficient in other elements of wellbeing, such as accomplishment and enjoyment. Moreover, they may even reasonably put their health at risk in order better to achieve some other aspect of wellbeing.

There is a further crucial point worth making about the individuation of the human right to health. Although many familiar human rights serve our interest in health in all sorts of important ways, this does not automatically render them components of the general human right to health. Yet, such an overly inclusive interpretation of the right to health has been advocated by the Committee on Economic, Social, and Cultural Rights, in its influential General Comment 14, as well as by other UN organs and leading global health scholars [22]. So, for example, Gostin notes that General Comment 14 treats as ‘integral components of the right to health’ entitlements to food, housing, life, education, privacy, and access to information. Gostin himself suggests that this specification is probably too ‘constrained’ and should be widened to include ‘gender equality, employment, and social inclusion’ [2, p. 257]. This inclusive approach is echoed, and perhaps taken even further, in a ‘Fact Sheet’ on the right to health jointly produced by the WHO and the Office of the UN High Commissioner for Human Rights. According to this document, the human right to health incorporates a slew of other rights, including gender equality and freedom from torture and other cruel, inhuman, or degrading treatment or punishment [23, p. 3]. By a process parallel to the WHO’s inflation of the notion of health to embrace all of human well-being, such interpretations appear to absorb within the human right to health all the rights that bear positively on our interest in health. Indeed, on this radically ‘inclusive’ approach, it is an open question, whether there is any right in the Universal Declaration of Human Rights, or in any of the two leading Conventions on Human Rights, which cannot be subsumed within the right to health, at least insofar as they involve duties that serve the right-holder’s interest in health. After all, a colourable story can be told of how denial of the rights to citizenship, political participation, a fair trial, freedom of speech, religion, movement, and association, among others, can have a seriously detrimental impact on health.

Now, something is clearly awry if the human right to health is lumbered with such a bloated interpretation.^7^ The mistake is to individuate the scope of the right simply by reference to whether a putative rights-based duty is justified, in part, by whether it serves a person’s interest in health. Many, if not most, human rights serve a person’s interest in health, and this is because they serve a multiplicity of interests, including health. Consider, for example, the fact that improvements in adult women’s education accounted for 40 % of the reduction in mortality between 1960 and 1990, although the steps taken to enhance educational provision are not obviously ‘health care’ measures [24, p. 94]. However, a human right is not picked out straightforwardly by the profile of interests it serves but, we claim, by reference to the subject matter of the obligations it generates.

More specifically, our suggestion is that the right to health should be construed as principally ranging over obligations concerned with the provision of health care services by medical professionals and public health measures, such as sanitation, potable water, clean air, alcohol, tobacco control, and so on. On this view, there is a moderate sense in which the human right to health is an ‘inclusive’ right. It ‘includes’, as justified components, various more specific rights to health care or public health measures, such as a right to health insurance or measles vaccination. By contrast, however, many so-called ‘social determinants of health’, which are crucial in promoting the health of individuals, do not come under the right to health. Instead, determinants such as education, housing, employment, and a social environment free of gender and racial discrimination—insofar as there is a right to them—more plausibly fall under other rights. The rationale for excluding these social determinants is partly a holistic one, turning on avoiding excessive overlaps with other rights there is good reason to recognise as distinct rights. But there is also a deeper rationale, which brings back the role of the interests served by the right in a more sophisticated manner. This is whether or not the object to which one has a right serves one’s interest in health as its primary and direct objective, as in the case of clean air and water, or whether it does so indirectly, via the serving of other interests which are its primary goal, as in the case of education and employment. Health care services and public health measures satisfy this criterion, but the social determinants of health typically do not.

One cannot, therefore, infer that the right not to be tortured or the right against degrading treatment are incorporated in the right to health, since these rights are not properly understood as having some specific connection with the provision of health care or public health measures.^8^ However, it is not always straightforward to draw clear lines between different human rights. Sometimes the boundaries will be blurred, and there will occasionally be tolerable overlaps in the scopes of rights. For example, the provision of training in first aid, or of health education more generally, might plausibly come under both the rights to health and to education. In consequence, it may sometimes be that the identical course of conduct constitutes a violation, or a fulfilment, of more than one human right. Often, laws will need to draw sharp lines between human rights so that overlaps can be avoided, where this would be beneficial in some way. We offer no general prescription for resolving these difficulties of line-drawing in a principled way, beyond the remarks about holism and primary and direct goals. What we have suggested, instead, is that the starting-point in delineating the human right to health has to be different from that adopted by the radical ‘inclusive’ view. That right, like all others, needs to be individuated by reference to the subject matter of the obligations associated with it.

It might be objected that the rejection of the radical inclusivity thesis expresses little more than a preference for tidy normative housekeeping. But this is not so: it also underwrites the idea that there are a number of fairly specific and irreducibly distinct human rights, so that enumerating a list of rights such as that in the Universal Declaration is a meaningful endeavour. It further caters to the idea that separating out various human rights is the best way of highlighting distinct normative concerns that might otherwise be obscured. It is worth underlining a significant practical pay-off of the approach we advocate. If one follows the radically ‘inclusive’ account to the right to health, then one faces a needlessly Herculean task when assessing the extent to which the right to health is being fulfilled globally. This is because the extent to which all health-enhancing rights are fulfilled will then need to be tracked. Progress towards such a massively sprawling goal is hard to monitor, and extremely difficult to achieve. This inevitably breeds uncertainty, frustration and despair. If one wishes to set a more determinate and manageable but still demanding task, then one should adopt the more constrained interpretation of the right to health.

It is clear, on the view we have developed above, that global health policy cannot be exclusively responsive to the right to health. This is so even if attention is confined to human rights that further the interest in health, as opposed to merely placing constraints on how it may be furthered. Other human rights are also extremely relevant, such as the rights to life, physical security, religious freedom, privacy, education, work, and so on. Indeed, as noted earlier, if one’s main concern is with the promotion of health overall, securing a right such as that of the right to education may be more important than other health-care related rights, such as the right to a minimum level of health insurance. Adopting an overly ‘inclusive’ interpretation of the right to health threatens to obscure the vital independent role these other rights must play in shaping global health policy.

## Content specification

The preceding discussion of the fallacy of radical inclusivity concerned mainly the individuation of the human right to health at an abstract level: the question of how it is to be distinguished from other human rights. But a deeper problem concerns the specification of its content, i.e., the content of the obligations associated with that right, even after their general subject matter has been identified. This is a difficult and many-sided topic, and here it is only possible to offer a few comments. Recall that a human right exists when, in the case of each human being, universal human interests generate obligations on others to respect, protect, and promote those interests in various ways. Obligations, or duties, are categorical and exclusionary reasons for action, non-compliance with which is a *pro tanto* basis for assigning blame (on the part of others) and experiencing guilt or self-blame (on the part of the duty violator).

It is a challenging task to articulate, in a general way, the conditions that need to be satisfied for the threshold from interests to duties to be crossed. However, at least two dimensions of this threshold are worth highlighting: *possibility* and *burden*. Both go some way towards unpacking the maxim ‘ought implies can’.^9^ On the one hand, an obligation will only arise when it is generally possible to comply with the counterpart duty in the case of all the supposed right-holders. The impossibility that prevents a duty arising may be of different sorts, ranging from logical impossibility to practical impossibility given fixed conditions of contemporary life. Taking an illustration of the latter kind, there may be inadequate resources now or in the foreseeable future to fulfil a proposed right for each individual human being. This would rule out, on any literal reading, a human right to health that imposed a duty to secure for all ‘the highest attainable standard of physical and mental health’.^10^ Understood as an absolute standard, it is impossible to bring all human beings to this very high standard, irrespective of the vagaries of their personal history and genetic constitution. But this supposed right is also ruled out by a more complex reason, even if what is ‘attainable’ is relativized to the history and genetic make-up of the individual right-holder. This is the fact that the state of one’s health depends not only on what others do but also on decisions made by the right-holder. It seems that the relativized right would require unacceptable interventions in the right-holder’s life, overriding their own health-affecting choices on matters such as diet, exercise, leisure activities, occupational choice, and so on. This would thwart the autonomy interests of the right-holder in ways that would preclude such a duty from arising. The content of the right might then be further weakened, so that it creates, for example, a duty to afford access primarily to the highest attainable standard of physical and mental *health care*, rather than health itself. This would leave the decision of whether or not to access such care largely to the discretion of the right-holder.

But even if a *pro tanto* case for such a demanding right to health could be made, it will very likely be defeated once the second dimension of the threshold is factored in. This is the dimension of burden, which registers the costs of affirming the right in relation to the interests of duty-bearers, the fulfilment of other human rights, as well as other values, such as respect for non-human nature. The right to health that arises from this process will, in all likelihood, be far less demanding than a right to the highest attainable standard of physical and mental health care, which is manifestly too costly. It is important to keep in mind, however, that ‘cost’ here is not a simple function of the real world market price of various medical services and public health measures. So, for example, one cannot simply take as given the market price that pharmaceutical companies, exploiting their market position and the rights afforded to them by intellectual property law, actually charge for their products. Equally, the cost cannot simply be a matter of the emotional strain and its consequences that compliance with the putative duty would entail, irrespective of the origin and nature of that strain. So, for example, the racist sentiments that make it ‘burdensome’, in some sense, for racists to conform with the rights of members of groups they despise do not bear on the question of whether members of those groups genuinely possess those rights. To take the racists’ burden into account would be to infect our thinking about which human rights people actually possess with their false beliefs about the relative moral standing of human beings. However, costs of both sorts might figure in deciding whether and how, all things considered, to insist on compliance with the rights in question.

The specification of the content of the human right to health is evidently a formidably complex matter. In this domain, as in others, a fully adequate specification through pure moral reasoning is unavailable. Instead, a workable standard must, to a large extent, be the product of social decision-making, whether conventional or legal. This is especially so when addressing the difficult questions of priority-setting in the use of limited resources. Of course, any such specification through social fiat must operate within tolerably determinate parameters set by moral reasoning.^11^ However, taking this line of thought much further, some theorists have responded to the problem of indeterminacy of content by appealing to a procedural criterion: the content of the human right to health will be largely fixed through fair processes of (in particular, democratic) decision-making.^12^ Of course, the question of what counts as a fair or democratic procedure is itself a contested matter. Bracketing that concern, it is still worth distinguishing at least two general roles for which such a procedure may be invoked.

The first is that of serving as a reliable epistemic guide to the actual content of the human right to health. But, if so, we need independent reasons for believing that procedures of the relevant sort are more likely to lead to correct specifications of that right than other procedures, such as judicial review or executive directives. Perhaps some of these reasons will be found in general epistemic virtues typically possessed by democratic institutions, such as inclusiveness in the range of interests and perspectives taken into account in law-making. However, it is doubtful that we can have confidence in a given decision-making process to specify the content of the right to health unless we have a prior, albeit incomplete, grip on the content of that right, and on pain of circularity, this grip cannot merely consist in the fact that the putative content was the outcome of that kind of process. In short, even granting its main premises, the epistemic appeal to procedure is by no means a comprehensive solution to the problem of content specification.

The second role decision-making procedures might perform is that of conferring legitimacy on any given legal specification of the right to health that they generates. Even if the content specified by law is not (obviously) correct as a matter of moral logic, that law may nonetheless be binding on its purported subjects in virtue, say, of its democratic pedigree. The first point to be made here is that the connection between procedure, including democratic procedures, and political legitimacy is not quite as straightforward as is often assumed. Contrary to a common view, it can be doubted whether democracy is generally either a necessary or a sufficient condition of legitimacy [29]. But the second, and more salient, point for present purposes is that the appeal to legitimacy effectively changes the subject. One is no longer addressing the original question—what is the content of the human right to health? Instead, one is asking a different question, namely, when is a law purporting to enact that right binding on its subjects? This latter question is certainly important, and it is also true that some of the indeterminacy surrounding the first question may be, as a practical matter, dispelled at the level of the second question. Nevertheless, in moving to the second question the focus has shifted from the requirements of justice to the legitimacy of law.

## Justice as the common good

Imagine a world in which the human rights of all people were fully realised. Could there nonetheless be grave health deficits in this world? The answer, it seems, is clearly ‘yes’. It follows, at least presumptively, that global health policy must attend to more than just securing people’s human rights.^13^ One potential health deficit in a human rights utopia is a high prevalence of obesity arising from the readily avoidable failure of people to maintain a healthy diet and exercise regimen. This could lead to severe health problems, but it would be a stretch to suppose that they are also necessarily human rights problems. Human rights are about how we treat others, not how we treat ourselves. In avoidably neglecting my health, I do not violate my own rights. On the contrary, I may be exercising my rights when I freely engage in unhealthy behaviour, such as smoking, over-eating, and avoiding exercise, knowing the risks and having viable alternatives. So, global health policy must be concerned with the reasons people have to promote their own health, including their duties to do so, and not just with human rights.

Another way health deficits might creep into a human rights utopia is if it is too demanding or intrusive for certain kinds of health-enhancing behaviour to be claimable as a matter of individual right. Consider someone in dire need of a kidney transplant. Although being given a matching kidney would certainly promote this person’s health interests, it is very doubtful that she has a right to another’s healthy kidney. This is because their interest in a kidney transplant is insufficient, by itself, to impose an obligation upon another to provide the organ for this purpose. Indeed, the right to bodily security stands in the way of others having a right to one’s kidney. Or consider participation in clinical trials. There are familiar difficulties in recruiting a sufficient number of trial participants in wealthier countries, something that in turn hampers valuable medical research. Yet, one normally should not suppose that anyone’s human right is being violated when people refrain from participating in clinical trials. Instead, it seems more natural to suppose that there is a human right of non-participation.

To clarify, we are not advancing the manifestly false thesis that obesity, organ donation, and research participation are utterly devoid of a human rights dimension. Certainly, people have a human right to such things as access to a healthy diet and treatment for obesity. But the incidence of obesity does not necessarily betoken a rights violation, something signalled by the fact that in the developing world, it is a condition more prevalent among those of a higher socio-economic status [32]. Also, there are presumably human rights-based obligations to facilitate organ donation and research participation and to offer or conduct them without discrimination, exploitation, or undue cost. But even when these demands are fully met, problems of obesity, lack of organs for transplant, and low research participation may nonetheless persist. Consequently, more than just human rights will be required to guide health policy in formulating and addressing these problems.

Global health policy must therefore include in its mission promoting compliance with various health-related norms, including duties to oneself and duties of charity, that are not claimable as a matter of human rights. Now, in the remainder of this article, we wish to outline a further type of ethical consideration—common goods, in particular, global common goods—that should also have an important place in global health policy. By ‘common good’, we do not mean aggregate social utility—the utilitarian notion of maximizing the aggregate welfare in society by means of a process of trading-off some people’s interests against those of others. Instead, according to a broadly Aristotelian interpretation, something qualifies as a common good if it serves the interests of all in a given community (universality), serves those interests in a uniform way for each person (uniformity), and does so in a non-rivalrous manner, i.e., the serving of anyone’s interests is not at the expense of serving any other’s (non-rivalrousness) [33].^14^ A shared language, such as English, is a common good so understood: it serves the interests of all in communication, it does so by furnishing all with a common means of communication, and one person’s use of English in no way detracts from anyone else’s capacity to draw on that language. In the health context, one can recognize the common good of a social ethos that both helps maintain an adequate supply of organs for transplant and ensures sufficient participation in valuable health-related research. Cultivating such a culture of compassion and participation goes beyond anything demanded by human rights, yet it is of great significance for the promotion of the health of all. The second sense of justice, distinguished at the outset, prominently includes duties to promote common goods.

The thrust of our argument so far has been that global health policy has to maintain a bifocal perspective insofar as justice is concerned: it has to be responsive both to human rights, including prominently the right to health, and to global common goods that bear on health. Now, Gopal Sreenivasan has recently expressed scepticism about the global health policy significance of the human right to health [36]. His claim is that, once common or public health goods are acknowledged, most of the requirements ordinarily thought to derive from the human right to health cannot be so understood. Instead, such requirements belong to the category of public (or common) goods. The argument proceeds on the basis that rights and common goods are mutually exclusive so that ‘no individual can have a moral claim-right to any pure public good’ [36, p. 256]. We can accept the assertion that much that is claimed under the heading of human rights involves securing a common good. But what justifies Sreenivasan’s contention that such claims therefore fail? In effect, his thesis—insofar as it bears on the position developed in this article—is that the relevant threshold from interest to duty is not satisfied. In particular, an individual’s interest in a public good, taken in isolation from others’ interests in that good, never suffices to impose a duty on others to deliver it. To take his example, securing the common good of herd immunity to diphtheria through a programme of vaccination involves the imposition of various burdens, not only the materials costs of the programme but also the ‘moral’ costs of compelling people to submit to it. In Sreenivasan’s judgment, it is ‘very doubtful that a single individual’s health has the moral significance to underwrite either cost, let alone both’ [36, p. 257].^15^

Sreenivasan’s novel argument merits attention for at least two reasons. The first is that it forces us to clarify the interest-based approach to human rights in order to explain more fully how interests generate duties and how the ensuing rights are related to the common good. Second, and just as importantly, Sreenivasan’s argument highlights an ambivalence within contemporary global health policy, one starkly illustrated by Gostin’s recent book, *Global Health Law*. Gostin is a prominent advocate of a human rights approach to global health, having taken the lead in calling for a global health framework convention grounded in the human right to health [2, pp. 437–439]. On the other hand, he also places great weight on public health measures and the social determinants of health, in contrast to medical services, partly on the grounds of their relatively greater preventive value [2, pp. 419–428]. But these considerations look like common goods, which may explain Gostin’s startling assertion, introduced without elaboration only fourteen pages shy of the conclusion of his treatise, that the right to health is not best seen as an individual right. Instead, he contends it is principally a ‘collective right’ that requires the implementation of broader public health and societal measures that are pre-conditions for securing more specific, individual rights [2, p. 426]. The radical idea that the right to health is a group right seems to presuppose something like Sreenivasan’s thesis that there can be no individual right to a common good. But this is a radical departure from the ordinary understanding of the human right to health, which is precisely that it is a right of individuals.

So, does Sreenivasan’s scepticism hold up? If his interpretation of when an individual’s interests suffice to generate a duty is correct, then his conclusion seems unassailable. After all, how could the health benefits of herd immunity for one individual, considered in isolation, justify the massive costs involved in instituting policies aimed at securing and maintaining herd immunity, such as compulsory vaccination? But the first indication that something is amiss here is that this pattern of argument generalizes alarmingly even to paradigmatic rights, such as the right not to be tortured. How could the benefits to any given individual of the criminal justice apparatus aimed at the prevention, detection, and punishment of torture, considered independently from the benefits to others, justify the massive costs of such a system? Indeed, it evidently follows that, on Sreenivasan’s approach, the morality of rights, including human rights, will justify *far fewer* entitlements than is ordinarily supposed. This is a conclusion drawn with alacrity by Allen Buchanan in a book that deploys Sreenivasan’s insight across a broad range of standardly acknowledged human rights [37].^16^

Rather than take Sreenivasan’s argument as revealing the severe limitations of rights morality, it should be seen as resting on a questionable interpretation of the threshold criterion for the emergence of human rights. For surely, no proponent of the interest-based view ever contemplated that the benefits to an individual alone sufficed to impose a duty to create and maintain vastly expensive public goods, such as a criminal justice system. Now, one response to this problem is to find ways in which the interests of others, rather than those of the right-holder, can work to justify the right to health, e.g., by pointing out that one’s interests are served *through* serving the interests of the right-holder [33]. However, we believe that although service to others’ interests may bear on the *weight* to be accorded to individual rights in practical deliberation, making them determinants of the very *existence* of those rights threatens to efface the distinction between the rights-based and non-rights-based parts of morality. Therefore, manoeuvres of this kind should not be the first line of response.

Instead, we simply reject the idea that on the interest-based account, the individual right-holder’s interest must suffice to justify a duty to bear the *whole* costs of the relevant public-good securing system. This ignores the fact that the right-holder is just one among many enjoying the benefits of the system. Just as the right-holder does not enjoy all of the benefits of a public good system, so too the justification of his right does not entail that his notional portion of the benefits justify the entirety of the cost. Instead, our contention is that what needs to be justified is the right-holder’s share of the costs among other right-holders who also benefit from the system. Given that we are dealing with human rights, and a standardized profile of interests that abstracts from certain variations among individuals, this share will be notionally the same for all. This notional equality applies *a fortiori* with respect to public health measures, which are typically not targeted at individuals and produce highly dispersed benefits. So, the real question posed by the threshold criterion is, does the benefit of herd immunity to any given right-holder justify a proportionate share of the costs involved in a vaccination programme aimed at securing it? An affirmative answer to this question is much more plausible than an affirmative answer to Sreenivasan’s question.

An immediate consequence of this view is that the relationship between human rights and common goods is not mutually exclusive. So, it is misleading for Sreenivasan to claim that ‘no individual can have a moral claim-right to any pure public good’. On the contrary, some *aspects* of the common good are rights-based, in the sense that they include elements to which we have a right. What these rights confer is a right to benefit from the common good in question [1, pp. 210–218]. An analogy may serve to illustrate this vital point. Compare two common goods in an academic department: a culture in which plagiarism is scrupulously avoided and, on the very few occasions on which it occurs, it is justly condemned and punished, and a culture in which academics adopt a friendly, highly collegial attitude towards one another. Both kinds of culture are common goods, meeting the requirements of universality, uniformity, and non-rivalrousness. But arguably, only the former is a common good that involves the securing of an individual right as part of its content. This is because the benefits to each individual of participating in a culture of anti-plagiarism have the significance needed to ground duties in others to undergo their proportionate share of the costs to generate those benefits. Now, something similar can be said about two health related common goods: the common good of herd immunity from diphtheria and the common good of a vibrant leisure culture. Both facilitate the health of members of the relevant community, but it is really only in the first case that the benefits of participation to the individual plausibly impose a duty on others to undertake their proportionate share of the costs to sustain the relevant common good.

## Conclusion

Human rights have a key role to play in global health policy. But more than human rights matter, and insofar as human rights matter, more than just the human right to health matters. We have argued against a normative monism in global health policy that operates only with human rights, or only with the human right to health, insofar as it engages with human rights. Instead, it is also important to factor in other ethical considerations, such as health-related duties to oneself and duties to foster health-related common goods, as well as human rights other than the human right to health. Moreover, the complex relations among human rights as they bear on global health policy, and between them and other aspects of the ethics of global health, need to be plotted in order to resist, in particular, the dogma that human rights and common goods are mutually exclusive and fundamentally antagonistic. In this way, human rights may be rescued from the distortions that they are liable to undergo at the hands of some of their most fervent and influential advocates in global health.

## Footnotes

1 For these two senses of justice, see Finnis’s discussion [1].

2 An important thrust of the human rights campaign in relation to the AIDS pandemic pioneered by Jonathan Mann was to reject ‘the prevailing view … that individual-centered human rights conflicted with community-oriented public health’ [2, p. 245]. This article aims to contribute to this attractive integrationist view by deepening its philosophical basis.

3 For a general account of the nature of human rights relied on here, see [6]. This article offers a critique of rival, ‘political’, interpretations of the concept of a human right offered by John Rawls, Charles Beitz, and Joseph Raz.

4 For a fine study of the human right to health, see [7].

5 This need not be an inevitable consequence of legalization; cf. the South African Constitutional Court’s decision in *Soobramoney v. Minister of Health (Kwazulu-Natal)* (1997), where it was held that provision of dialysis for a patient with chronic renal failure was not required by the constitutional right to health, partly because this would prejudice the satisfaction of other health needs that have to be met out of the state’s budget [15].

6 For agency and needs-based accounts of human rights, see [11, 19].

7 The upshot is so peculiar that one might wonder why radical inclusivism is so popular. Mindy Roseman has suggested (in conversation) that it is sometimes viewed as a way of upholding the credentials of the human right to health against those who are sceptical of ‘socio-economic’ human rights, by showing that it incorporates traditional civil and political rights. Whatever its efficacy at the level of rhetoric, however, this strategy offers no real defence of the positive primary duties associated with the right to health.

8 For a similar interpretation of ‘the human right to health care’, see [25, pp. 205–206]. However, the authors erroneously suppose that the human right to health exclusively reflects our interest in health [25, p. 206].

9 For a fuller account of the threshold, see [17].

10 See article 12 of the International Covenant on Economic, Social and Cultural Rights [26].

11 On the process of *determinatio* through which law gives specific content to objective moral requirements, see [1, pp. 284–289].

12 Daniels, for example, has characterised the human right to health as “implying entitlements that individuals have to a socially relative array of services (in the case of healthcare) that is the outcome of a process of fair deliberation under reasonable resource constraints” [27, p. 134]. The point is developed with regard to priority setting in [28]. For the claim that democratic states should be largely exempted from the authority of international human rights institutions when it comes to specifying the content of the human right to health, at least with respect to their own citizens, see [25].

13 The idea that global health policy should be exclusively grounded in human rights (and in the human right to health in particular) animates the proposal for a framework convention on global health advanced by Gostin et al. [30, 31].

14 Economists go further and add a condition of non-excludability, i.e., it is impossible to exclude others (cheaply) from enjoying public goods if they are provided at all; see the helpful discussion in [34]. Although non-excludability is particularly relevant in addressing issues of self-interested incentives to contribute to the maintenance of a common good, we take as our focus the wider notion. As O’Neill shows, an emphasis on non-excludability makes it especially difficult to establish the existence of *global* common or public goods. Hence her suggestion that it is preferable to focus on goods with dispersed benefits, the weaker definition of public goods adopted in the UNDP-sponsored [35].

15 This is Sreenivasan’s attack on the variant of the argument for a right to a public good that invokes an interest-based account of rights. He also has an argument against the possibility of a right to a public good on a will-based account of rights. We find the latter persuasive, given the inconsistency of many public goods, e.g., herd immunity, with the comprehensive individual opt-outs (waivers of rights) that a will-based theory would require.

16 The supposed broader pay-off for Buchanan is that international human rights law, which contains these ambitious requirements, is not best understood as ‘mirroring’ a background morality of human rights. Insofar as our argument works against Sreenivasan’s claim, it also works against the legal implication Buchanan seeks to draw from it.
